# Fractionated irradiation of right thorax induces abscopal damage on testes leading to decline in fertility

**DOI:** 10.1038/s41598-019-51772-y

**Published:** 2019-10-23

**Authors:** Junling Zhang, Dan Yao, Yimeng Song, Yan Pan, Lin Zhu, Yang Bai, Yanwu Xu, Jianghong Zhang, Chunlin Shao

**Affiliations:** 1 Institute of Radiation Medicine, Shanghai Medical College, Fudan University, Shanghai, 200032 China; 2 Department of Biochemistry, College of Basic Medicine, Shanghai University of Traditional Chinese Medicine, Shanghai, China

**Keywords:** Apoptosis, Infertility

## Abstract

Radiation-induced abscopal effect (RIAE) may influence radiotherapy efficiency. However, it is unknown whether RIAE triggers abnormal genetic consequence. We present a novel evidence that, when mice were given fractionated irradiation on right thorax, the ultrastructure of blood-testis barrier was damaged in company with apoptosis induction in testes, and the sperm number and vitality were drastically decreased so that both the fertility and the survival of their offspring were reduced. Protein microarray assay and hormone detection showed that some cytokines especially TNF-α, TGF-β and estradiol in the serum of irradiated mice increased to higher levels in consistent with abscopal damage, and this conditioned serum had toxic effect on TM4 cells *in vitro*. When the mice were fed with cimetidine, the above abscopal responses were significantly attenuated. This study demonstrates in the first time that the thoracic irradiation (Th-IR) induces structural and functional damage in the distal testes and further cause fertility decline of irradiated male mice, which may have important implications in the strategy development of radiotherapy in avoiding abnormal genetic consequence.

## Introduction

Radiotherapy is one of the main methods of cancer treatment. However, ionizing radiation (IR) could inevitably damage normal tissues during radiotherapy that usually induce side effects on patients such as bone marrow suppression and even has a probability of secondary cancer incidence^1–3^. Many clinical case reports have shown that the cell and tissue injuries can be induced in the organs distant from irradiated tumor site, which is referred as radiation-induced abscopal effect (RIAE)^4–7^. For example, RIAE occurs on 4 of 28 patients with renal carcinoma after the treatment of stereotactic ablative radiotherapy, in three of these four cases, the untreated metastatic tumors are completely regressed without relapse from two to four years after radiotherapy^8^.

Most previous literatures about RIAE focused on the regression of nonirradiated metastatic lesions after localized tumor radiotherapy. However, RIAE is a double-edged sword and it may cause serious side effects on normal tissues^9,10^. When the left-half side of mouse body is irradiated with 1 Gy of X-rays, nearly 6 h after IR, the single- and double-strand breaks of DNA are detected in the skin cells of the right side^11^. IR on the lower half of the body of mouse could even induce tumor development in the nonirradiated head, which provides direct experimental evidence of the carcinogenic effect of RIAE^12^.

Since RIAE has important implications in radioprotection and radiation safety, the molecular identities of signaling factors and underling mechanisms are very attractive but they are remaining in poor understanding. Enormous evidence shows that the soluble factors such as NO, ROS, CO, TGF-β1, COX-2, interleukins and cytochrome-c are the important signals of radiation-induced bystander effect (RIBE) that could cause a serious of biological endpoints including DNA damage, gene instability, cell apoptosis, gene mutation and even tumorigenesis^9,13–17^. Recently, it has been found that the cysteine protease CPR-4 can be secreted from irradiated Caenorhabditis elegans into the conditioned medium that further cause bystander responses in the unirradiated animals^18^.

Radiotherapy is a main treatment method of lung cancer and it could stimulate acute responses such as inflammation and pneumonitis and even induce chronic effects of pulmonary fibrosis^19^. During this radiotherapy, the normal tissue of lung will be unavoidably irradiated, but so far it is unknown whether this radiation damage could induce any harmful abscopal effects on distal tissues. For the first time, this work presents that the Th-IR on male mice induces abscopal damage in the distal testes and further yields genetic consequence of fertility decline. The potential signaling factors involved in this RIAE are also explored.

## Results

### Thoracic irradiation (Th-IR) induces tissue damage in testes

**Figure 1:**
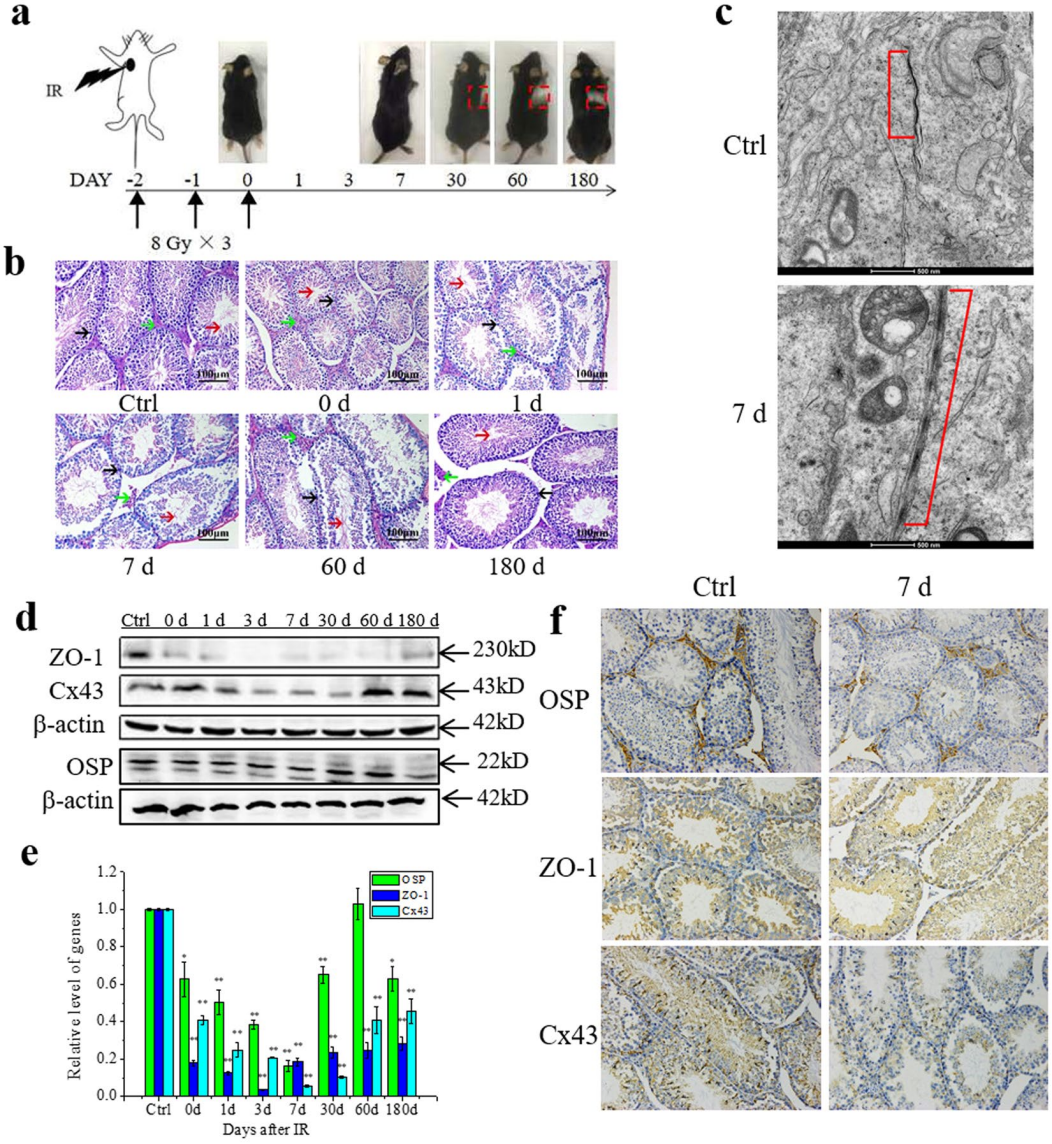
Right Th-IR induced distal damage in mice testes (n = 7). (**a)** The schema of fractionated irradiation. Mice were irradiated in a field of 1 × 1 cm^2^ on right thorax with 8.0 Gy each time at day -2, -1 and 0 and then sacrificed for further measurements at day 0, 1, 3, 7, 30, 60 and 180 after IR. (**b)** The histological images of testis tissue sections. Blank arrows, spermatogonia; red arrows, sperms; green arrows, basement membrane. (**c)** Representative ultrastructure images of BTB (red bracket) in the testis tissue at day 7 after IR. Images were obtained with a perspective electron microscope. Scale bars, 500 nm. (**d)** The expressions of ZO-1, OSP and Cx43 proteins in the testis. The proteins were measured by Western blotting. (**e)** The relative mRNA levels of *ZO-1*, *OSP* and *Cx43* gene in the testis. The mRNAs were measured by qRT-PCT. (**f)** Immunocytochemistry staining of ZO-1, OSP and Cx43 proteins in testis tissues at day 7 after IR. *P < 0.05 and **P < 0.01 compared with corresponding control. Depict cropped blots obtained by each protein evaluation. Full-length blots are shown in the Supplementary Fig. S1.

After 30 days of IR, the mouse hair in the radiation area became sparse and faded to white, indicating that the location of radiation field is precise (Fig. 1a). At different time points after Th-IR, the mice testes were collected for histological observation and molecular analysis. As shown in Fig. 1b, the pathological structures of seminiferous tubules were damaged immediately (day 0) and these injuries were maintained at least for 3 months after Th-IR. In addition, the spermatogenic cells became disorder and even sloughed (black arrow), the gap (green arrow) between seminiferous tubules increased, and the number of sperm (red arrow) in the uterine cavity of seminiferous tubules decreased. On day 180 after Th-IR, the arrangement of spermatogenic cells was reorganized to normal status, but the gap between seminiferous tubules was still larger and the number of sperm cells was also less than the control group.

The blood-testis barrier (BTB) is a physical barrier between blood vessels and seminiferous tubules in animal testis and it is constructed by tight junctions, adherens junctions and gap junctions between two Sertoli cells. When this barrier is destroyed, the cytotoxic chemical in the serum can pass through and cause testis damage. A representative image of the ultrastructure of BTB in Fig. 1c shows that the BTB was lined on the Sertoli-Sertoli cell interface (seeing the red bracket in the upper panel). However, after Th-IR, the BTB was partly broken and its tight junctions became disintegrated and this BTB damage had the highest degree on the 7th day after Th-IR (the lower panel in Fig. 1c).

The disorder of BTB structure may result from the alterations of its regulation proteins. Hence, we checked the expression of BTB structure related genes and proteins of ZO-1, OSP and Cx43 by qRT-PCR and Western blot (WB), respectively. Figure 1d,e illustrate that, the levels of mRNAs and proteins of these genes changed in a time-dependent manner and had minimum values at day 7 and then recovered gradually, but they were still lower than those in the nonirradiated control at day 180 after IR. To further confirm these phenomena, we measured these proteins *in situ* in the testes tissue by immunochemistry assay. The representative images in Fig. 1f show that the proteins of ZO-1, OSP and Cx43 were mainly located around the testicular basement membrane. At day 7 after Th-IR, the expressions of these proteins in the testis of irradiated mice were much lower than that in the control group.

### Th-IR induces cell apoptosis in distal testis

**Figure 2:**
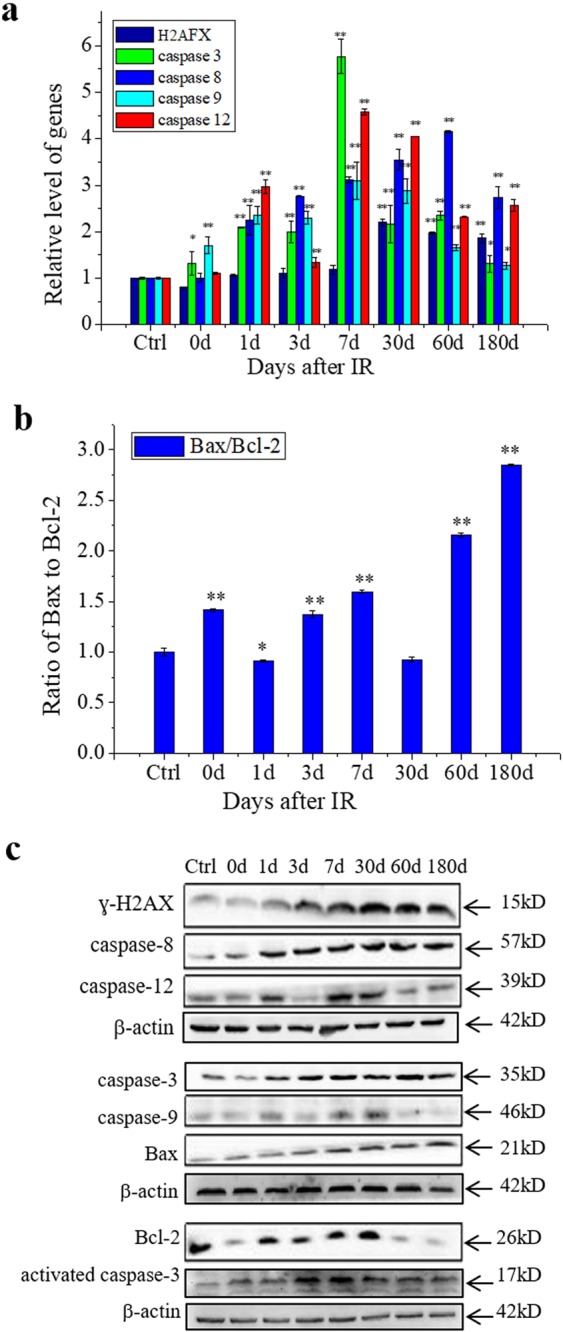
Inductions of apoptosis and related genes and proteins in the testis tissue at indicated time points after Th-IR (8 Gy × 3) (n = 7). (**a)** Relative expression levels of *H2AFX*, *caspase-3*, *caspase-8*, *caspase-9* and *caspase-12*. (**b)** Ratio of *Bax/Bcl-2* gene expression. (**c)** Expressions of ɣ-H2AX, activated caspase-3, caspase-3, caspase-8, caspase-9, caspase-12, Bcl-2 and Bax proteins detected by Western blot assay. *P < 0.05 and **P < 0.01 compared with corresponding control. Depict cropped blots obtained by each protein evaluation. Full-length blots are shown in the Supplementary Fig. S2.

To further make sure the occurrence of RIAE, we detected DNA damage and apoptosis induction in testis. The phosphorylation of histone H2AX is a rapid cellular response to DNA damage. Figure 2a illustrates that, the mRNA level of *H2AFX* in the testis tissue was significantly increased to about 2-times of control from day 30 to 180 although it was not increased in the first 7 days. Meanwhile, the expressions of apoptosis-related genes of *caspase-3*, *caspase-8*, *caspase-9* and *caspase-12* in testis were up-regulated in a time-dependent manner i.e., they increased along with the time after Th-IR and approached to the highest levels at day 7 and then gradually decreased until day 180 (Fig. 2a). In addition, the expression of pro-apoptotic gene of *Bax* was increased while the anti-apoptotic gene of *Bcl-2* had opposite changes so that the ratio of *Bax/Bcl-2* increased in the testis after Th-IR (Fig. 2b). The above alterations of gene expressions were confirmed by the WB assay of corresponding proteins (Fig. 2c).

### Th-IR causes oligospermia and dysfunction of mice fertility

**Figure 3:**
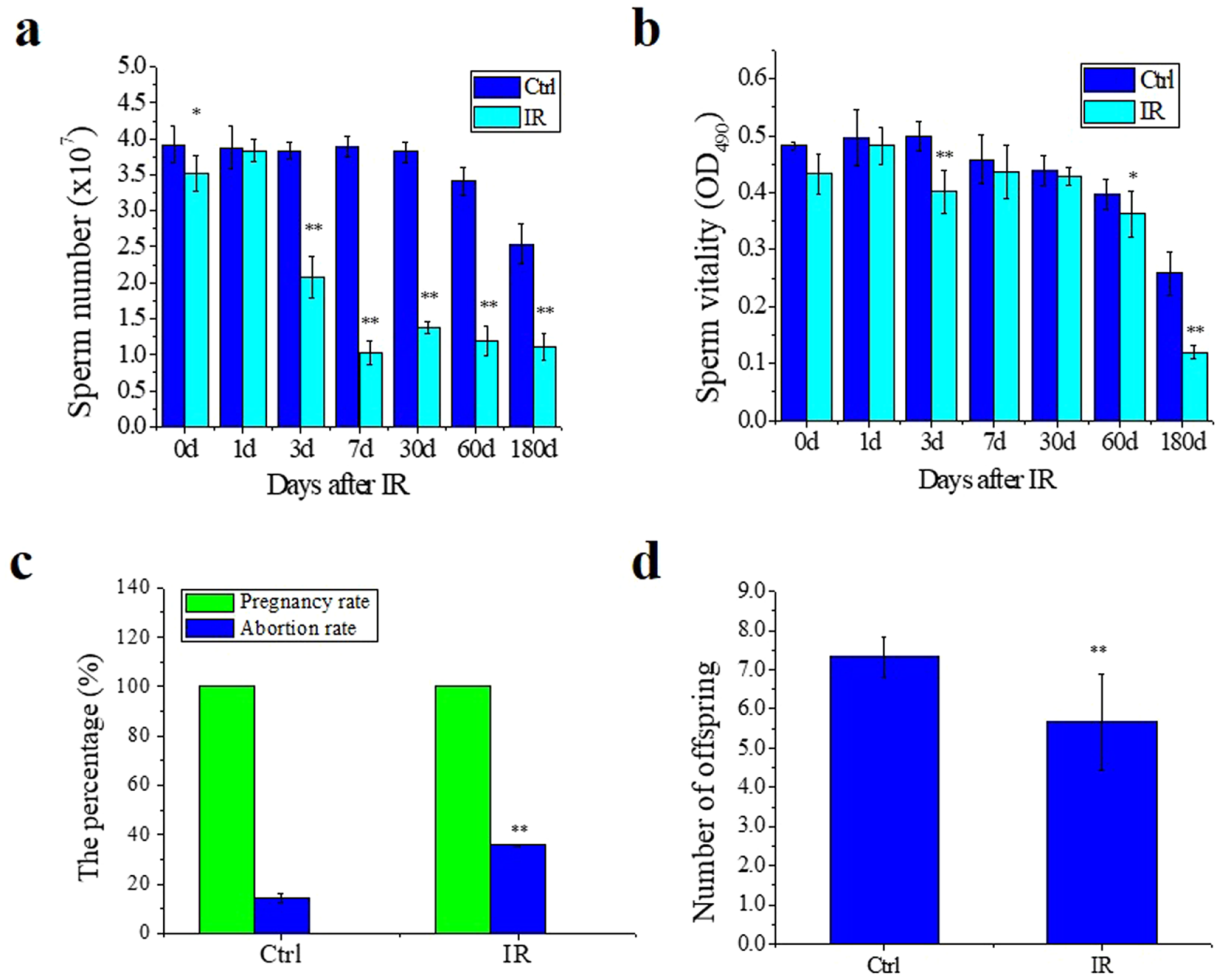
Th-IR (8 Gy × 3) causes abscopal injuries in sperm cells and decline in fertility (n = 7). (**a)** Sperm number of male mice with and without Th-IR at different time points after IR. (**b)** Sperm vitality of male mice with and without Th-IR at different time points after IR. (**c)** The pregnancy rate and abortion rate of female mice. **(d)** The average number of above offspring in one generation. *P < 0.05 and **P < 0.01 compared to the corresponding control.

Sperm is one of the most sensitive cells to inflammatory infection^20^. A result shows that an oligospermia phenomenon occurred after Th-IR. In comparison with nonirradiated control, the number of sperms in the thoracic irradiated mice was drastically decreased from day 3 and kept at even low levels from day 7 to day 180. (Fig. 3a). Meanwhile, the sperm vitality in the distal testis was significantly reduced especially at day 3 and day 180, although the sperm vitality of the control mice had a decrease tendency along with its age (Fig. 3b).

A fateful consequence of the decreases of sperm vitality and sperm number in the testis is the reduction of fertility ability. At 7 days after Th-IR, the irradiated male mouse and nonirradiated female mouse were caged together pair-by-pair for mating. Pairs of nonirradiated male and female mice were served as control. We found that the abortion rate (the offspring dead on the day of birth) of the IR group was about 2-times of nonirradiated control although the Th-IR of male mouse didn’t influence the pregnancy rate of its paired female mouse and the pregnancy rates of both control and Th-IR groups were 100% (Fig. 3c). Moreover, the average number of offspring in the Th-IR group was lower than that of the control group (Fig. 3d). These results give direct evidence that Th-IR of male mice induces a genetic abscopal effect of fertility decline.

### Th-IR breaks the balance of sex hormones

**Figure 4:**
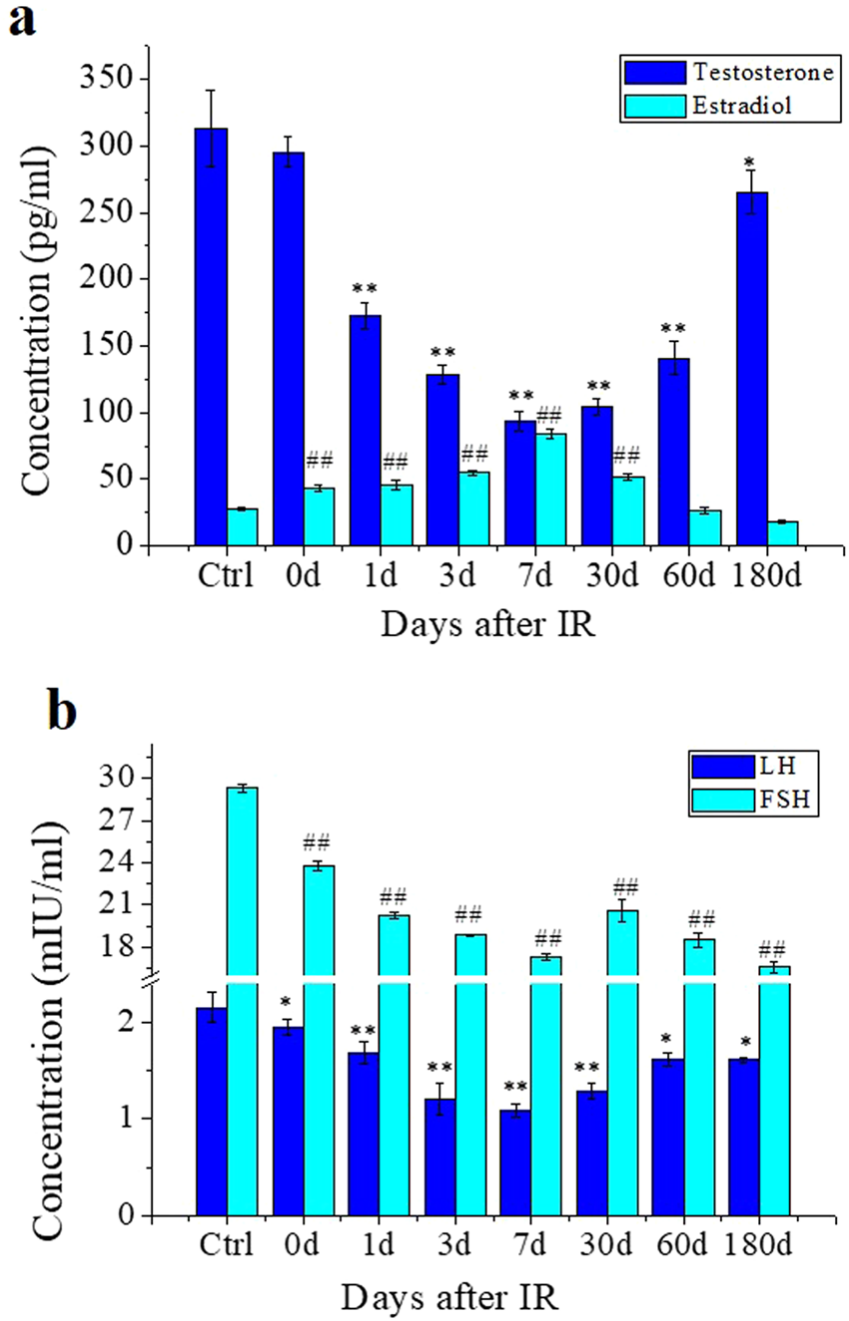
Concentrations of sex hormones in the serum of mice with fractionated irradiation (8 Gy × 3) on right thorax (n = 7). The serum was collected at different time points after IR. **(a)** The concentrations of testosterone (T) and estradiol (E2) in the serum. (**b)** The concentrations of luteinizing hormone (LH) and follicle stimulating hormone (FSH) in the serum. * and ^#^*P* < 0.05 compared with corresponding control. ** and ^##^*P* < 0.01 compared with corresponding control.

The Th-IR induced abscopal testis damage and genetic effect should have close relationship to radiation-induced signaling factors. Hormones and inflammatory factors are two important potential objectives since their abnormal changes in endocrine system may cause reproductive dysfunction^21–26^. Here we detected these factors in mouse serum before and after fractionated irradiation on right thorax and the results showed that the levels of testosterone (T) and luteinizing hormone (LH) obviously decreased from day 1 to the lowest values at day 7 and gradually increased but still less than control until day 180 after IR (Fig. 4a,b). The content of follicle stimulating hormone (FSH) in the serum had a similar time-response to T and LH after IR (Fig. 4b). In contrast, the level of estradiol (E2) increased since day 0 after IR and it had the highest value at day 7 after IR and then gradually returned to control level at day 180 after IR (Fig. 4a). These time-responses are consistent with that of RIAE of testis tissue damage where the BTB injury and the sperm cell apoptosis are most remarkable at day 7 after IR.

### Cytokines induced by Th-IR

**Figure 5:**
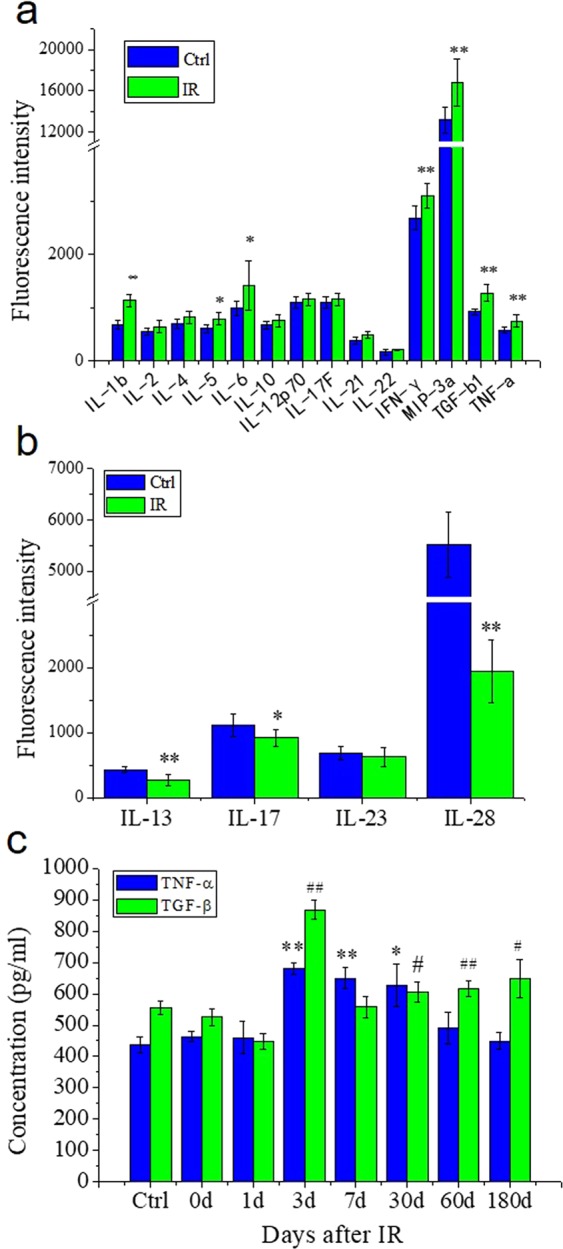
The relative expression levels of differential cytokines in the serum of mice with fractionated irradiation (8 Gy × 3) on right thorax (n = 7). (**a)** Up-regulated cytokines at 7 days after Th-IR. (**b)** Down-regulated cytokines at 7 days after Th-IR. (**c**) The time-response of the concentrations of TNF-α and TNF-β after Th-IR. * and ^#^*P* < 0.05 compared with corresponding control. ** and ^##^*P* < 0.01 compared with corresponding control.

To know any cytokines being involved in the RIAE, a total of 18 cytokines in mouse serum were detected with a microarray kit. The results showed that, after Th-IR, 7 cytokines (IL-1β, IL-5, IL-6, IFN-γ, MIP-3α, TGF-β1, TNF-α) were significantly up-regulated (*p* < 0.05) (Fig. 5a), and 3 cytokines (IL-13, IL-17 and IL-28) were significantly down-regulated (*p* < 0.05) (Fig. 5b). Among these upregulated cytokines, TGF-β and TNF-α had the highest increasement ratio (IR group/Ctrl group) of 1.38 and 1.29, respectively. It has also been reported that these two cytokines could induce BTB damage and cell apoptosis^27–29^. Thus we further measured the time-response of them after Th-IR. Figure 5c illustrates that the concentration of TNF-α in mice serum drastically increased from day 3 after Th-IR and kept at high levels until one month and then decreased to control level in 2–6 months after Th-IR. The concentration of TGF-β increased to a quite high level at 3 days and maintained at high level from 1 to 6 months after Th-IR. It is noticed that both inflammatory cytokines of TNF-α and TGF-β approach to their highest levels on the day 3 after Th-IR.

### Cimetidine relieves Th-IR induced abscopal effects on testis

**Figure 6:**
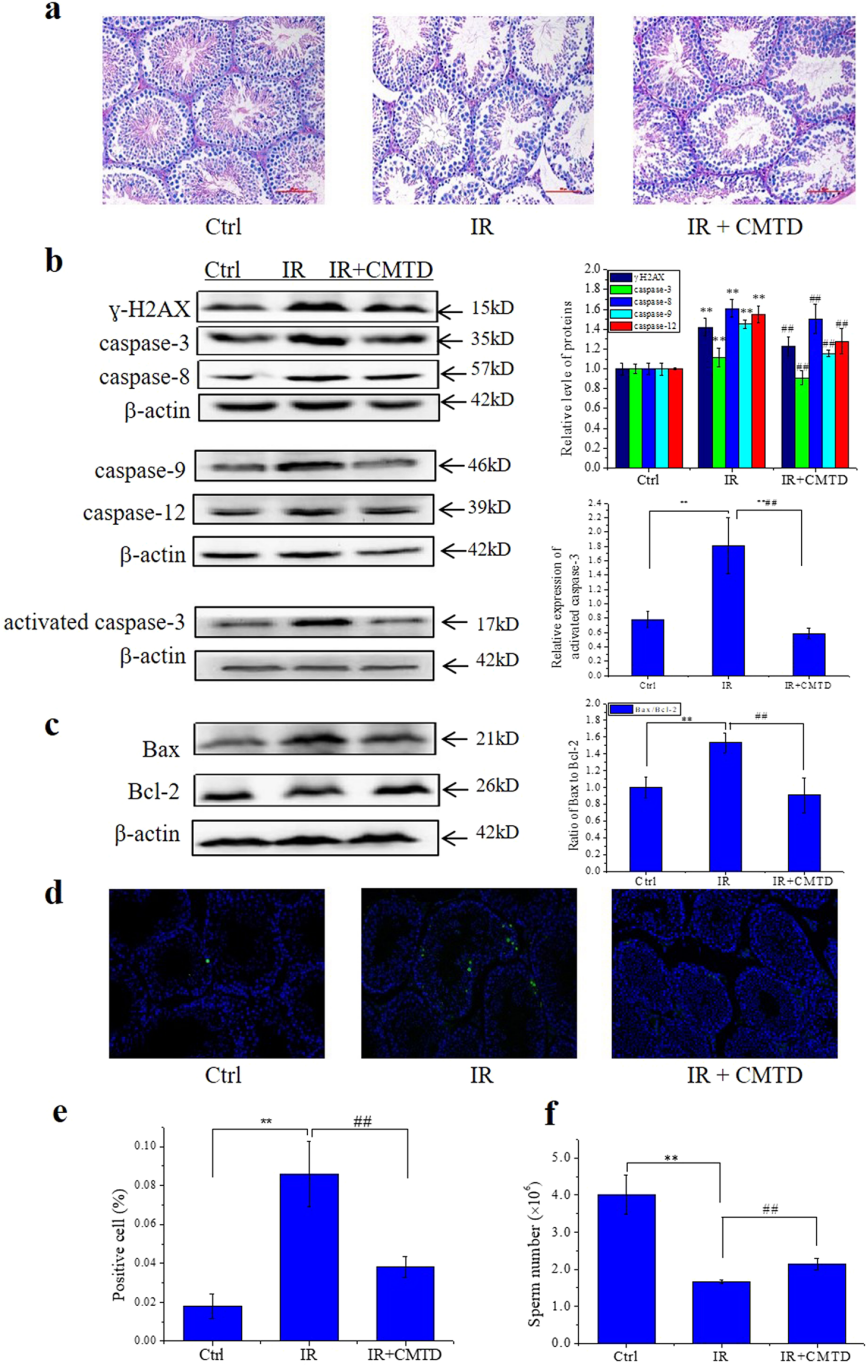
Treatment of mice with cimetidine (CMTD) relieves Th-IR induced abscopal effects (n = 7). After fractionated irradiation (8 Gy × 3), the mice were treated with CMTD by means of intragastric administration for 7 days and then sacrificed for further analyses. (**a)** HE staining of the mice testis tissue sections of IR and IR + CMTD groups. (**b)** Expressions of apoptosis related proteins of ɣ-H2AX, activated caspase-3, caspase-3, caspase-8, caspase-9 and caspase-12 and their relative levels in mice testes. (**c)** Expression of Bax and Bcl-2 proteins and their ratio in the mice testes. Depict cropped blots obtained by each protein evaluation. Full-length blots are shown in the Supplementary Fig. S3. (**d**,**e**) The Tunnel images of apoptotic cells and the apoptosis rate in testis tissues (200×). (**f**) The sperm number in the epididymis of mice. *P < 0.05, **P < 0.01 compared with Ctrl. ^##^*P* < 0.01 compared with IR.

**Figure 7:**
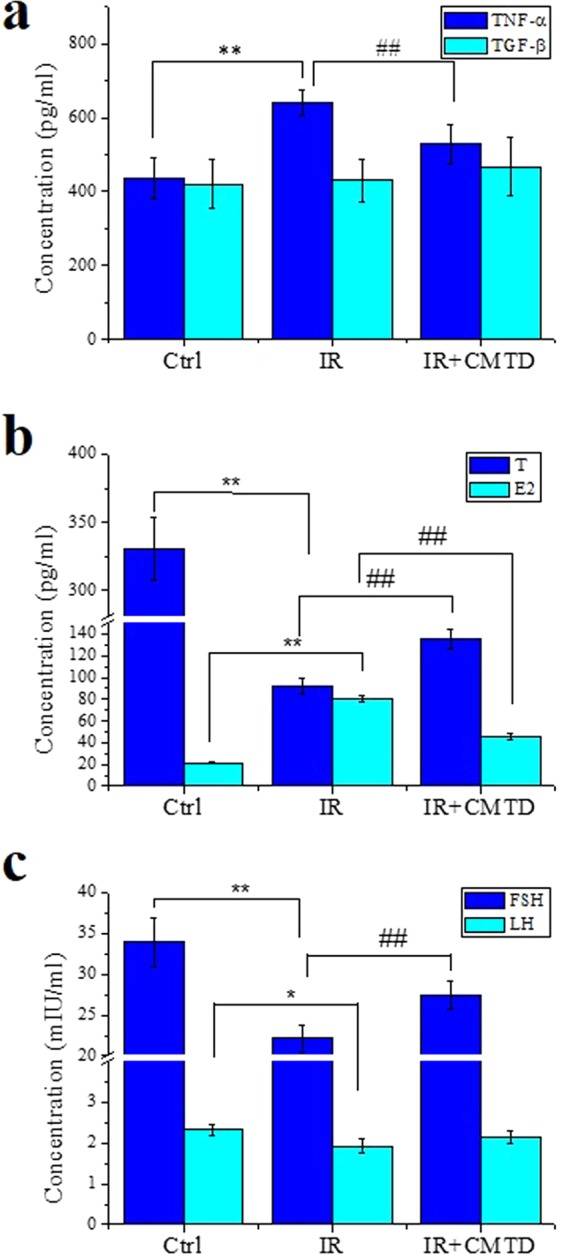
Treatment of mice with cimetidine (CMTD) relieves Th-IR induced sex hormone abnormal and inflammatory reaction (n = 7). (**a)** The concentrations of TNF-α and TGF-β in mice serum. (**b)** The concentrations of T and E2 in mice serum. (**c)** The concentrations of FSH and LH in mice serum. *P < 0.05, **P < 0.01 compared with Ctrl. ^##^*P* < 0.01 compared with IR.

The above findings indicate that the inflammatory factors may be involved in the RIAE. To further confirm this deduction, we treated the male mice with cimetidine (CMTD) by means of intragastric administration after Th-IR since CMTD is an anti-inflammatory drug^30^. Figure 6 illustrates the representative results of the effect of CMTD on RIAE at 7 days after Th-IR. This drug treatment effectively attenuated the structure damage of distal testes in the mice with Th-IR (Fig. 6a). Compared with the radiation alone group, the spermatogenic cells were arranged neatly and the gap between seminiferous tubules became compact, and the expression levels of apoptosis-related proteins were partly reduced (Fig. 6b) and the Bax/Bcl-2 ratio was diminished (Fig. 6c) in the testes tissue of the IR + CMTD group. TUNEL assay showed that few apoptotic cells were detected in the seminiferous tubules of nonirradiated mouse testis. After Th-IR, the number of the apoptotic spermatogonia cells located at the outermost layer of the seminiferous epithelium of the testis was increased and approached to the highest level at day 7 after IR, but this increase was diminished by CMTD treatment (Fig. 6d,e). As an important consequence of the drug intervention, the sperm number in the epididymis of the IR + CMTD group was effectively recovered to a level higher than that of the IR group (Fig. 6f). Moreover, the treatment of mice with CMTD attenuated radiation-induced upregulation of TNF-α and E2 and downregulation of T and FSH, but it had little influence on TGF-β and LH in the mice serum at day 7 after Th-IR (Fig. 7a–c).

### Conditioned serum induces apoptosis in TM4 cells *in vitro*

**Figure 8:**
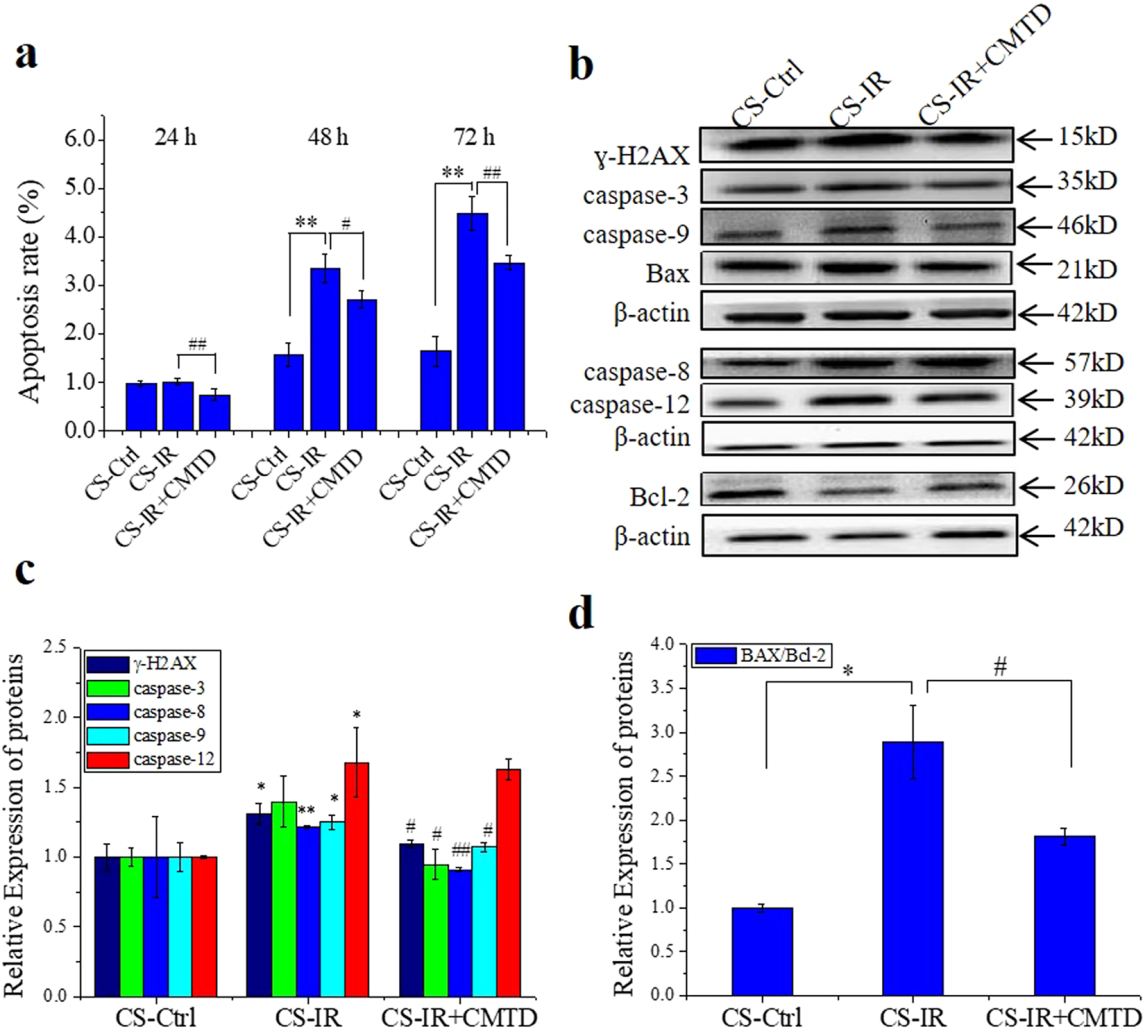
Effects of the conditioned serum (CS) on TM4 cells *in vitro*. The CS was collected from the mice without irradiation (CS-Ctrl), with fractionated irradiation (8 Gy × 3) on right thorax (CS-IR), and treated with CMTD for 7 days after above irradiation (CS-IR + CMTD). TM4 cells were cultured with 10% of these CS for 24, 48 and 72 h, respectively. **(a)** TM4 cell apoptosis induction after different time of CS treatment. (**b**–**d)** Apoptosis-related protein expressions of ɣ-H2AX, caspase-3, caspase-8, caspase-9, caspase-12, Bax, Bcl-2 and their relative levels in TM4 cells after 72 h of CS treatment. The proteins were detected by Western blot assay. *P < 0.05 and **P < 0.01 compared with CS-Ctrl. ^#^*P* < 0.05 and ^##^*P* < 0.01 compared with CS-IR. Depict cropped blots obtained by each protein evaluation. Full-length blots are shown in the Supplementary Fig. S4.

The generation of RIAE indicates that, when the mouse is irradiated through thorax, some active signaling factors should be released into its serum and transported by blood to affect distal organs. We wonder whether this serum is toxin enough to induce cellular damage. For this purpose, we collected the conditioned serum (CS) from the male mice with or without Th-IR and then cultivated TM4 Sertoli cells with 10% of this CS for different times from 24 to 72 h. It was found that the cell apoptosis was significantly induced by the CS from irradiated mice (CS-IR) after 48 h of cell culture and the apoptosis rate was continually enhanced after 72 h of cell culture (Fig. 8a). When the mice were fed with CMTD, the toxic effect of the serum (CS-IR + CMTD) on apoptosis induction of TM4 cells was obviously reduced in comparison with that of CS-IR. Further measurements showed that, after 72 h of cell culture with CS-IR, the expressions of γ-H2AX protein and caspase proteins in TM4 cells were increased differently (Fig. 8b,c), and the Bax/Bcl-2 ratio was increased to 3-times of CS-Ctrl (Fig. 8b,d). Meanwhile, when the irradiated mice were treated with CMTD, the effects of its serum (CS-IR + CMTD) on the expressions of these apoptosis-related proteins were reduced in comparison with that of CS-IR (Fig. 8b–d).

## Discussion

To our knowledge, this study demonstrates for the first time that Th-IR induces distal damage in testes including morphological structure damage, apoptosis induction, sperm validity decreases, and even yields genetic damage of fertility decline. A novel finding is that, after Th-IR, the Sertoli cells are severely vacuolated, the BTB is partly destroyed to be disconnected and the expressions of BTB-related proteins are down-regulated. It is well known that BTB not only prevents the macromolecules in serum from entering the seminiferous tubules but also prevents the essential substances from leaving the seminiferous tubules in the processes of spermatogenesis. This selective function of BTB creates a unique microenvironment for postmeiotic spermatid development in mammalian testes during the seminiferous epithelial cycle of spermatogenesis. If this structure is destroyed, the microenvironment of spermatogenesis will be imbalanced^31^ and further influence normal physiological function of spermatogenic cells that are highly susceptible to a various of endogenous and exogenous stimulus^32^. Therefore, the BTB structure damage may be an important reason of the significant decreases of sperm number and sperm viability after Th-IR.

The reproductive system damage and spermatogenic structure disorder may cause grievous consequences such as infertility^33–36^. We found here when the male mice are given Th-IR, the quality and survival of their offspring are reduced significantly. The fertility has close connection with male reproductive endocrine function that is mainly regulated by hypothalamus, pituitary and gonads. The hypothalamus could regulate the release of FSH and LH through secreting gonadotropin-releasing hormone. FSH and LH can promote sperm maturation and androgen generation. E2, the predominant form of estrogen, also plays a critical role in male sexual function and it is essential for modulating libido, erectile function and spermatogenesis^37^. It has also reported that E2 could decline the testicular testosterone synthesis^38^, and the administrating of exogenous estrogens exerts detrimental effects on mouse testis and interferes with intra-testicular steroidogenesis^39^ and finally induces Sertoli cells apoptosis. Although the mechanisms involved were not clear, human patients with oligoasthenoteratozoospermia and oligozoospermia had higher testicular E2 concentrations than patients with normozoospermia^40^. Furthermore, the increased E2 and reduced T concentrations were present in the seminal plasma of patients with azoospermia. Therefore, estradiol is closely related to testosterone and sperm number. Here we find that the levels of some hormones are obviously changed in the irradiated mice (Fig. 3). The levels of T, LH and FSH have a time-response opposite to E2 and they have the lowest levels at the day 7 after Th-IR. Decreased T level is clearly associated with low libido in male. In addition, it was reported that T could promote the integrity of BTB *in vivo*^41,42^ and *in vitro* by enhancing the recycling of internalized proteins to the cell surface and relocating these proteins to reassemble and seal the barrier^43,44^. Accordingly, the BTB damage associated with hormone changes might have a negative effect on male endocrine function and hence influence male fertility.

Our data demonstrate that the testis tissue damage and related molecular changes have the most serious situation at the day 7 after Th-IR. This time window is consistent to the induction of E2 and TNF-α and TGF-β that have the highest levels after 7 d of Th-IR, which gives a clue that these factors might contribute to the above abscopal responses. In fact, it has been reported that the BTB dynamics during spermatogenesis are regulated at least in part by cytokines that determines the steady-state levels of integral membrane proteins at BTB^45^, and the local administration of TNF-α to testes disrupt the BTB integrity reversibly^46^.

Cytokines play an important role in innate immunity, apoptosis, angiogenesis, cell growth and differentiation. Cytokines are also involved in the process of most diseases including cancer^47,48^ and reproduction disorders^23,49,50^. It has reported that proinflammatory cytokines could affect BTB permeability and enter into the seminiferous tubules to induce apoptosis of germ cells^51^. Our results of cytokine assay showed that, after Th-IR, the levels of IL-1β, IL-5, IL-6, IFN-γ, MIP-3α, TGF-β1, TNF-α were significantly increased in the mice serum, and among them TNF-α and TGF-β had the highest increasement ratio.

TNF-α is a pro-inflammatory cytokine and plays complex roles in radiation injury^52^ and contributes to apoptosis induction^53–55^. TNF-α can activate the Fas-TNFR associated death domain protein and further activate caspase-3 and caspase-8 leading to apoptosis eventually^56^. This work shows that the content of TNF-α in serum increased in parallel with the over expressions of a series of apoptosis-related proteins in testis tissue from day 3 to 30, indicating that TNF-α may contribute to the RIAE.

On the other side, TGF-β is a well-known factor that can be released from irradiated lung tissue and is an important signaling factor in RIBE of DNA damage and cellular apoptosis^57,58^. TGF-β can regulate a variety of cellular processes in testes including the secretory function of Sertoli cells, the testicular development and spermatogenesis^59,60^, and it acts on cellular tight junction by regulating the expression of OSP^61^. We have found TGF-β participate RIBE both *in vivo* and *in vitro*^62,63^ and contribute to fractionated thoracic irradiation induced abscopal damage on rat testes. This study shows that TGF-β is overexpressed in the serum of irradiated mice and maintains at high level from day 3 to 180 after Th-IR accompanying with the increases of apoptosis and tissue damage in testis, indicating that TGF-β may also be involved in the abscopal testis damage after Th-IR.

CMTD is a potent histamine H_2_ receptor antagonist and clinically used in the treatment of ulceration of stomach and intestine without obvious side effects. Recently, it is reported that CMTD has protective effect against radiation damage of fast neutron and γ-rays^64,65^. The current results manifest that CMTD could effectively attenuate the distal damage induced by Th-IR, and it also reduces the cytotoxic effect of CS from irradiated mice on cell apoptosis *in vitro*. The regulation ability of CMTD on RIAE should result from its anti-inflammatory function^30^, which is demonstrated in Fig. 5 that the treatment of mice with CMTD reduces radiation-induced generation of TNF-α in mice serum.

Taken together, this study gives direct evidence that the localized Th-IR not only induce abscopal damage in the testes but further reduces the fertility of male mice. The abscopal damage on testes is attenuated by CMTD, indicating the inflammatory factors such as TNF-α and TGF-β and hormones may be involved in the RIAE. Although the precise molecular mechanisms of these distal effects on genital system are still not clear and need to be investigated intensively, the current discovery has important implications in developing new strategy of radiation protection during radiotherapy for young male patients to avoid any abnormal genetic consequence.

## Methods

### Mice and treatments

Six-week-old C57BL/6 J male mice (18–20 g, Certificate number: 20170005000867) were purchased from Sino-British SIPPR/BK Lab. Animal Co. Ltd (Shanghai, China) and housed for one week before experiments and maintained at a constant temperature (25 °C) with 12 h light and dark cycles. These mice were randomly divided into control group and IR group with 7 mice for each treatment. For the IR group, mice were locally irradiated through the right thorax (1 × 1 cm^2^) by three fractionated doses of X-rays (8 Gy per IR in 3 days) at a dose rate of 0.883 Gy/min (X-RAD 320, PXI Inc., North Branford, CT, USA; 12 mA, 2-mm aluminum filtration). At present, a large fractionated dose is being investigated as a new strategy of radiotherapy. Some animal studies have shown that 8 Gy × 3 is an ideal irradiation protocol to yield significant anti-tumor abscopal effect after tumor irradiation^66,67^.

In some experiments, mice were provided with cimetidine (CMTD, 100 mg/kg·d) by means of intragastric administration for 7 days starting from the day of the final irradiation. It has been reported that CMTD has a radioprotective effect by antioxidation and immunomodulation^64,68,69^. All animal experiments were approved by the Animal Ethical Committee of Fudan University and all methods were carried out in accordance with the guidelines of animal welfare legislation.

### Mouse serum and tissue collection

The mice were sacrificed on day 0, 1, 3, 7, 30, 60 and 180 after the last irradiation, respectively. The mice serum was isolated. Mice testes were quickly picked up and fixed for hematoxylin and eosin (HE) staining, TUNEL apoptosis and immunofluorescence analyses. All serum and testes were frozen and stored at −80 °C until use.

### Testis histology

The fixed testes were dehydrated and embedded in paraffin then cut into 3–5 μm thickness of tissue sections. These sections were deparaffinized and stained with HE. The morphologic changes of the testes were observed under a light microscopy (Nikon, Tokyo, Japan).

### BTB integrity assay

The testis tissue was immediately picked and spliced into 1–2 mm^3^ pieces using a double-edged blade and fixed in the glutaraldehyde solution. Then tissue pieces were rinsed with 1% osmium tetroxide in phosphate buffer and dehydrated with an ethanol gradient, embedded in gelatin capsules and made into ultra-thin (70–80 nm) sections on a grid overnight, and then dried with 3% uranyl acetate-lead citrate for 15–20 min. Finally, the tissue images were acquired by a perspective electron microscope (Tecnai-G2-F30, FEI, Portland, USA).

### TUNEL assay

The paraffin-embedded testes sections were stained with a TUNEL kit *in situ* (Roche, Basel, Switzerland). The apoptotic cell images in the tissue were captured with a fluorescence microscope (DFC450-C Leica, Wetzlar, Germany).

### Immunohistochemical staining

After being deparaffinized, rehydrated and quenched, the tissue sections were incubated with the primary antibodies of ZO-1, Cx43 (1:200, Proteintech, Rosemont, IL, USA) and OSP (1:200, Signalway Antibody, Maryland, USA) and subsequently treated with appropriate second antibody (1:5000, Signalway Antibody). Then the tissue sections were stained by diaminobenzidine, hematoxylin and observed with a light microscope (200×).

### Quantitative real-time PCR assay

Total cellular RNA and transcripts (1 μg RNA/sample) were extracted with a commercial kit (Takara, Dalian, China) and analyzed by a qRT-PCR System (LightCycler® 96, Roche, Basel, Switzerland). The GAPDH RNA was used as an internal standard. The qRT-PCR primers used are shown in Supplementary Table S1. The gene expressions were calculated by the comparative CT method.

### Western blot assay

Total protein was extracted from frozen testis tissues using RIPA buffer and protease inhibitors. Proteins were fractionated by 10% SDS-PAGE. The membranes were blocked in TBST solution (5% milk in TBST with 0.05% Tween-20) and incubated with primary antibody overnight at 4 °C. The primary antibodies were used for ɣ-H2AX, caspase-8 (1: 1,000, Cell Signaling Technology, Danvers, MA, USA), caspase-3, caspase-9, caspase-12, Bax, Bcl-2 and β-actin (1:1000, Proteintech). Then, the membrane was incubated with appropriate second antibody (1:10000, Signalway Antibody) and visualized using an enhanced chemiluminescence detection kit (Millipore Corp., Massachusetts, USA).

### Sperm number and vitality assay

At different time after Th-IR, the epididymal cauda was isolated from the mice testis and the connective tissue and blood vessels were carefully removed. Sperm suspensions were prepared by mincing cauda in 1 ml phosphate-buffered physiological saline (PBS). The suspension was pipetted and filtered through 80 μm nylon mesh to remove tissue fragments. A solution containing the sperm was then transferred to the chamber of a hemocytometer. And sperm counts were manually monitored and counted by light microscopy (Olympus, Japan). The result is expressed as sperm count/ml. Sperm vitality was measured by 3-(4,5-dimethylthianol-2-yl)-2,5-diphenylte-trazolium bromide (MTT) colorimetric reduction assay. The vitality was measured by measuring the absorbance at 570 nm (relative to the 630 nm reference) using microplate reader (Infinite M200Pro, Tecan, Switzerland).

### The fertility and offspring survival assay

Seven days after Th-IR, one male mouse from the IR group and one female (C57BL/6 J, 18–20 g) mouse without any treatment were caged together for mating. Each group had ten pairs of mice. The pregnancy rate and abortion rate of female mice, the average number of offspring were recorded.

### Microarray analysis for cytokines

Cytokines in mice serum were detected by a protein array according to the manufacturer’s manual (Ray Biotech, Inc., USA). This assay is a multiplex sandwich enzyme-linked immunosorbent assay (ELISA)-based system that includes a membrane spotted with 18 selected cytokine- or chemokine-specific antibodies (including IFN-γ, IL-1β, IL-10, IL-12 p70, IL-13, IL-17A, IL-17F, IL-2, IL-21, IL-22, IL-23 p19, IL-28A, IL-4, IL-5, IL-6, MIP-3α, TGF-β1,TNF-α). Each sample was measured in triplicate. InnoScan 300 Microarray Scanner (Innopsys, Brabrand) was used to collect fluorescence intensities.

### Detection of sex hormones and inflammatory factors

The hormones, TNF-α and TGF-β in the serum were detected by commercial ELISA kits. The kits for T and E2 were from Cayman (Michigan, USA). The kits for FSH and LH were from Enzyme-linked Biotechnology (Shanghai, China). The kits for TNF-α and TGF-β were from Neobioscience (Shenzhen, China). The sample absorbance was recorded by the microplate reader (Infinite M200Pro, Tecan).

### Measurements of cellular effects of CS

TM4 cells, a mouse Sertoli cell line obtained from the ATCC, were cultured in DMEM/F-12 medium supplemented with 10% serum (5% horse serum and 5% fetal bovine serum) and maintained at 37 °C in a humidified atmosphere with 5% CO_2_. The serum from CS-Ctrl, CS-IR and CS-IR+ CMTD were collected from mice at 7 days after Th-IR. To test the cytotoxic effect of these serums, the TM4 cells were cultured with a medium contained 10% CS for 24 h, 48 h and 72 h, then cell apoptosis was detected by flow cytometry, the apoptosis-related proteins were measured by WB.

### Apoptosis detection

Cell apoptosis was detected by flow cytometry using an Annexin V FITC Apoptosis Kit (556547, BD, USA). Briefly, TM4 cell was treated by conditioned medium. Afterward, the cells were collected, washed triply with PBS, and strained with 5 μl of Annexin-V-FITC in 100 μl binding buffer for 20 min at room temperature in the dark. After washing, 10 μl propidium iodide in 100 μl binding buffer were added to the cell suspension and incubated for 10 min at room temperature in the dark. Cell apoptosis was scored by quantifying the population of Annexin V-FITC-positive cells for 10,000 events and analyzed by flow cytometry (CytoFLEX, Beckman, USA).

### Statistical analysis

Data were expressed as mean ± SD of 3–5 independent experiments and analyzed with One-way ANOVA method using the SPSS17.0 software (SPSS Inc., Chicago, IL, USA). *P* < 0.05 was considered as significant difference between the indicated groups.

## Supplementary information

Supplementary table and figures
