# Supplementary material for: Fractionated irradiation of right thorax induces abscopal damage on testes leading to decline in fertility
Source: Sci Rep. 2019 Oct 23;9:15221. doi: 10.1038/s41598-019-51772-y (PMC6811594; doi:10.1038/s41598-019-51772-y)
Supplement: Supplementary file 1 — Supplementary table and figures [file 41598_2019_51772_MOESM1_ESM.docx]

**Supplementary Information**

# Fractionated irradiation of right thorax induces abscopal damage on testes leading to decline in fertility

**Running title:** Thoracic irradiation induces testes damage and fertility decline

**Authors:**

Junling Zhang^1^, Dan Yao^1⸸^, Yimeng Song^1^, Yan Pan^1^, Lin Zhu^1^, Yang Bai^1^, Yanwu Xu^2^, Jianghong Zhang^1*^, Chunlin Shao^1*^

⸸ 1These authors contributed equally to this work.

***Authors for correspondence:**

Prof. Chunlin Shao, Phone: +86-021-64048677, E-mail: [clshao@shmu.edu.cn](mailto:clshao@shmu.edu.cn)

Dr. Jianghong Zhang, Phone: +86-021-64436075, E-mail: zjh551268@fudan.edu.cn

**Affiliations:**

^1^Institute of Radiation Medicine and School of Basic Medical Sciences, Fudan University, Shanghai 200032, China.

^2^Department of Biochemistry, College of Basic Medicine, Shanghai University of Traditional Chinese Medicine, Shanghai, China

**Supplementary Table S1: List of primers used in this study.**

| **Gene** | **Forward primer** | **Reverse primer** |
| --- | --- | --- |
| *GAPDH* | GCC TTC CGT GTT CCT ACCC | TGA AGT CGC AGG AGA CAA CC |
| *Bax* | CAG AGG ATG ATT GCT GAC G | GTC CCG AAG TAG GAG AGG AG |
| *Bcl-2* | GCT ACC GTC GTG ACT TCG C | CCC AGC CTC CGT TAT CCT |
| *Caspase-3* | TCT GAC TGG AAA GCC GAA AC | ACT GGA TGA ACC ACG ACC C |
| *Caspase-8* | TCG TCT ATG GAA CGG ATG G | TCT CGG TAG GAA ACG CAG T |
| *Caspase-9* | GGC TGT TAA ACC CCT AGA CCA | TGA CGG GTC CAG CTT CAC TA |
| *Caspase-12* | TAG GGG AAA GTG GCA GTT TCA | GGG CCA ATC CAG CAT TTA CCT |
| *H2AX* | TGG AAA GGG TCA GGG AAC G | GAC TTG TGC TGG TAT CTG GGT G |
| *ZO-1* | ACC ACC AAC CCG AGA AGA C | CAG GAG TCA TGG ACG CAC A |
| *OSP* | CGG GCA TTC CAT TGT TGT T | AAG GCT TCC ACT GTC GTT G |
| *Cx43* | CTT CCC TGG CCT GAC CCT CT | GCT TGC CTC CCT GAT GCT AA |

Figure S1


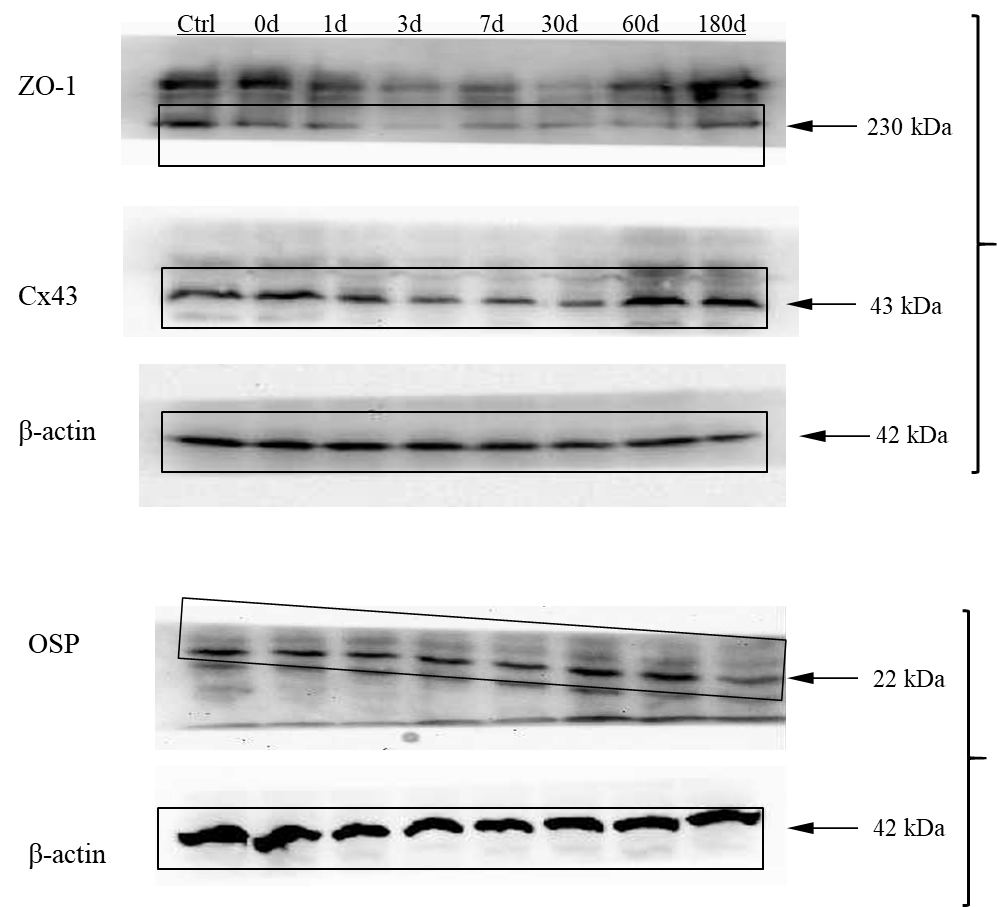


1. Note: means that they come from the same member.
2. Note: means that they come from the same member.

Figure S2


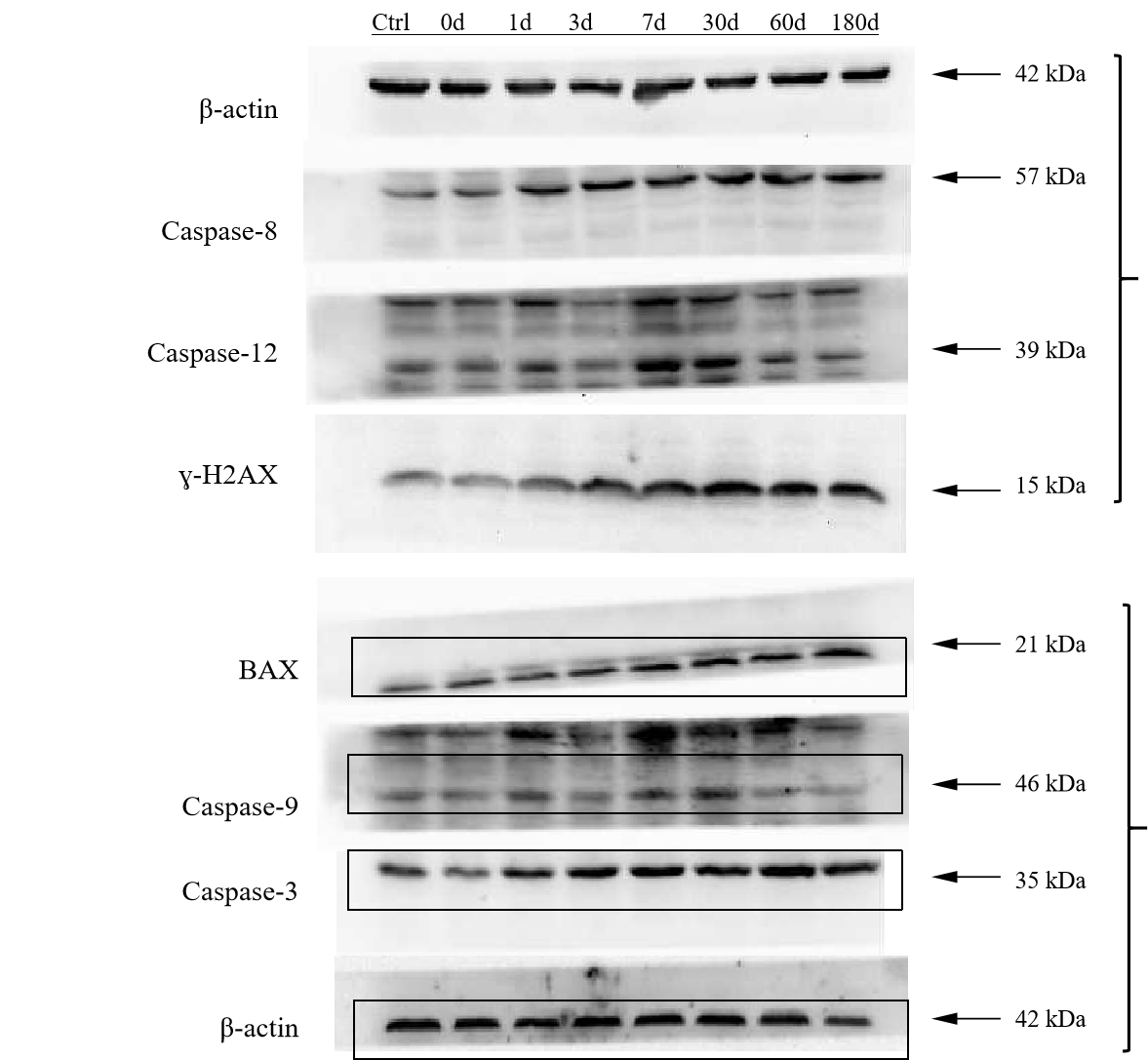


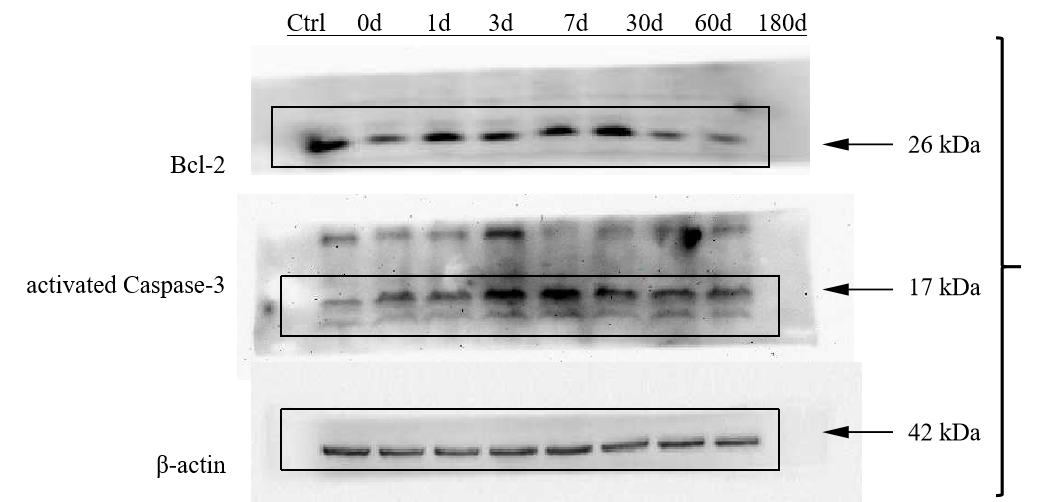


1. Note: means that they come from the same member.

Figure S3


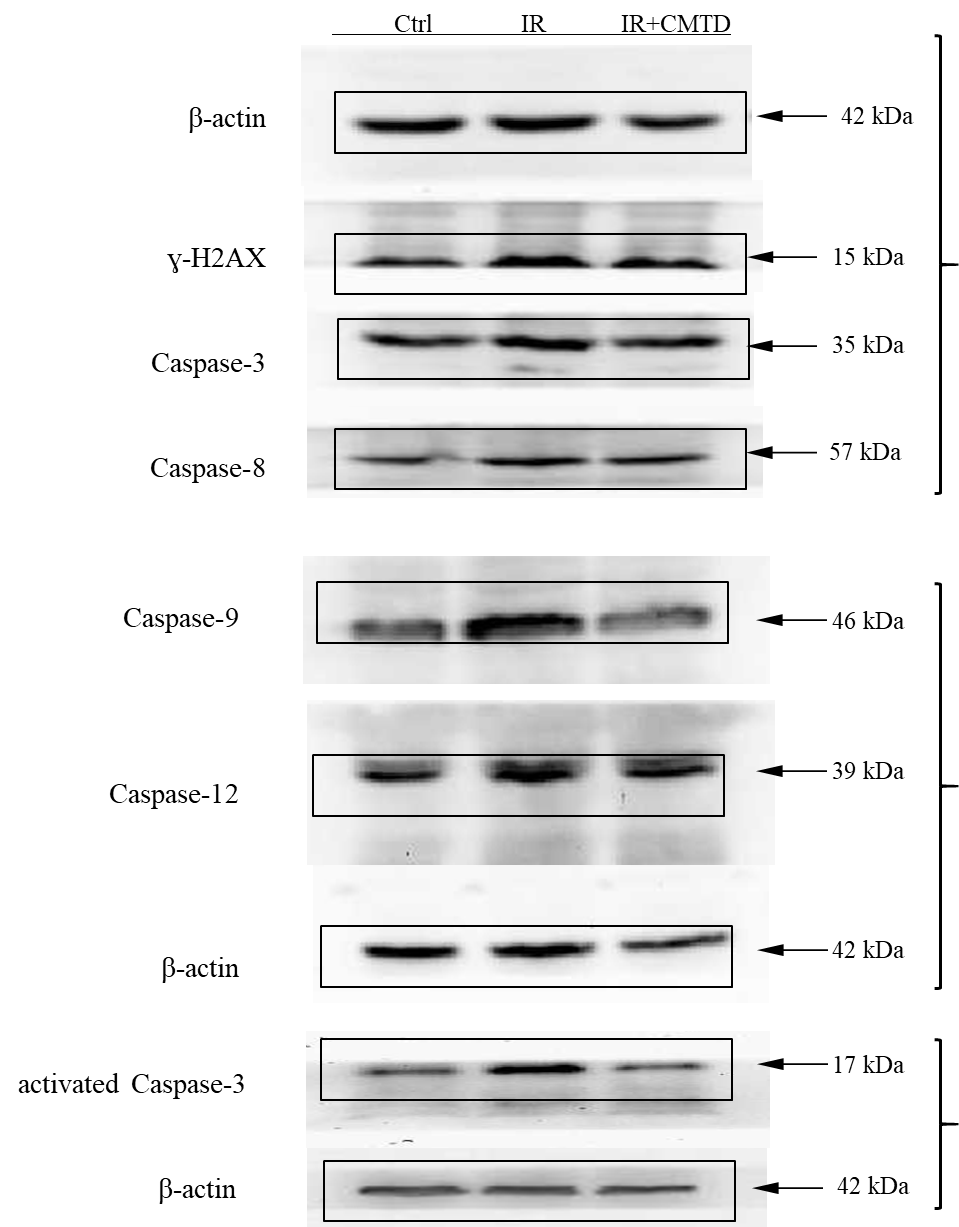


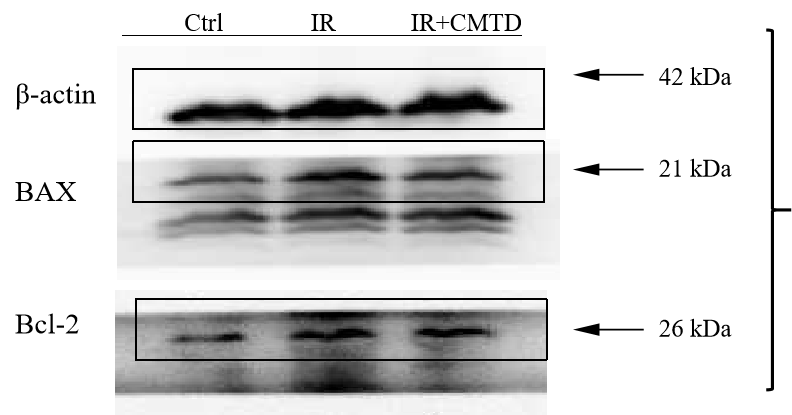


1. Note: means that they come from the same member.

Figure S4


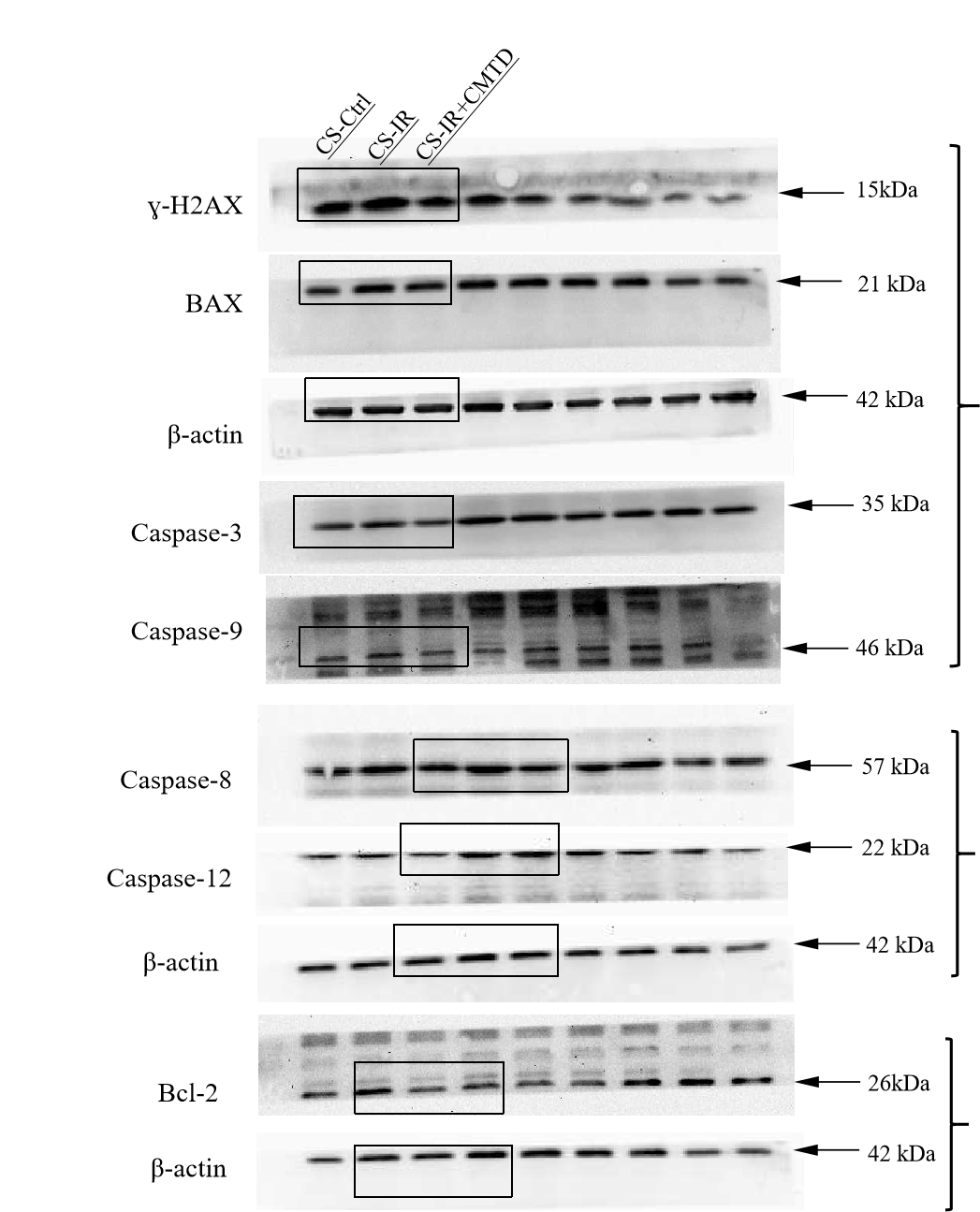


1. Note: means that they come from the same member.
